# Spaceflight increases sarcoplasmic reticulum Ca^2+^ leak and this cannot be counteracted with BuOE treatment

**DOI:** 10.1038/s41526-024-00419-y

**Published:** 2024-07-19

**Authors:** Jessica L. Braun, Val A. Fajardo

**Affiliations:** 1 Department of Kinesiology, Brock University, St. Catharines, ON Canada; 2 Centre for Bone and Muscle Health, Brock University, St. Catharines, ON Canada

**Keywords:** Calcium channels, Physiology

## Abstract

Spending time in a microgravity environment is known to cause significant skeletal muscle atrophy and weakness via muscle unloading, which can be partly attributed to Ca^2+^ dysregulation. The sarco(endo)plasmic reticulum Ca^2+^ ATPase (SERCA) pump is responsible for bringing Ca^2+^ from the cytosol into its storage site, the sarcoplasmic reticulum (SR), at the expense of ATP. We have recently demonstrated that, in the soleus of space-flown mice, the Ca^2+^ uptake ability of the SERCA pump is severely impaired and this may be attributed to increases in reactive oxygen/nitrogen species (RONS), to which SERCA is highly susceptible. The purpose of this study was therefore to investigate whether treatment with the antioxidant, Manganese(III) *meso*-tetrakis(*N*-n-butoxyethylpyridinium-2-yl)porphyrin, MnTnBuOE-2-PyP^5+^ (BuOE), could attenuate muscle atrophy and SERCA dysfunction. We received soleus muscles from the rodent research 18 mission which had male mice housed on the international space station for 35 days and treated with either saline or BuOE. Spaceflight significantly reduced the soleus:body mass ratio and significantly increased SERCA’s ionophore ratio, a measure of SR Ca^2+^ leak, and 4-HNE content (marker of RONS), none of which could be rescued by BuOE treatment. In conclusion, we find that spaceflight induces significant soleus muscle atrophy and SR Ca^2+^ leak that cannot be counteracted with BuOE treatment. Future work should investigate alternative therapeutics that are specifically aimed at increasing SERCA activation or reducing Ca^2+^ leak.

Exposure to microgravity, and subsequent muscle unloading, is known to cause extensive muscle weakness and atrophy, especially to postural muscles such as the soleus^1–6^. Recent work has demonstrated that muscle weakness precedes muscle atrophy^4^ and was thought to be due, at least in part, to Ca^2+^ dysregulation^7^. Using soleus muscle samples from the rodent research (RR) -1 and -9 missions, we demonstrated the Ca^2+^ uptake ability of the sarco (endo)plasmic reticulum Ca^2+^ ATPase (SERCA) pump to be severely impaired following ~1 month of spaceflight^5^. In muscle, SERCA is responsible for maintaining low intracellular Ca^2+^ concentrations ([Ca^2+^]_i_) by bringing Ca^2+^ from the cytosol into its storage site, the sarcoplasmic reticulum (SR)^8,9^. Impaired SERCA function can result in high [Ca^2+^]_i_, leading to elevated RONS, increased protein degradation, cell death, and muscle weakness and atrophy^10–14^. Structurally, SERCA pumps are highly susceptible to post-translational modifications (e.g., tyrosine nitration, cysteine nitrosylation) from elevated RONS that will impair its catalytic activity ^5,15–22^. The ensuing elevated [Ca^2+^]_i_ will not only lead to muscle damage but can also further increase RONS production^23–25^ creating a negative cyclic relationship that perpetuates muscle pathology.

While damaging at high concentrations, RONS are important signaling molecules at low concentrations^26^. Superoxide dismutases (SOD) are a family of enzymes that maintain physiological RONS concentrations by scavenging and neutralizing highly reactive superoxide molecules (for review, see ref. ^27^). Mice deficient of SOD show impaired SERCA activity, muscle weakness, and atrophy^11,20,22^; but, the pharmacological activation of SERCA can reduce mitochondrial RONS production and attenuate muscle atrophy despite increased oxidative stress^11,12^. Thus, targeting SERCA, either through genetic overexpression or pharmacological intervention, may be a viable target to attenuate muscle atrophy and weakness. The purpose of this study was therefore to investigate whether treatment with Manganese(III) *meso*-tetrakis(*N*-n-butoxyethylpyridinium-2-yl)porphyrin, MnTnBuOE-2-PyP^5+^ (BuOE, also seen as BMX-001), an antioxidant that acts as a SOD mimetic^28,29^, could attenuate muscle atrophy and SERCA dysfunction in the murine soleus muscle following 35 days of spaceflight. BuOE has been effective in reducing oxidative stress in various animal models of radiation-induced injury^30–33^, a relevant injury mechanism to spaceflight, providing strong rationale for the investigation of its effectiveness in the present study.

Following 35 days of exposure to microgravity, absolute soleus weight was significantly reduced in the flight groups compared to GC/VIV groups (*p* = 0.0005) with no effect of BuOE treatment (Fig. 1A). No effects of flight or treatment were observed on body weight (Fig. 1B) resulting in a main effect of flight reducing the soleus:body weight ratio (*p* = 0.0053, Fig. 1C).

**Fig. 1:**
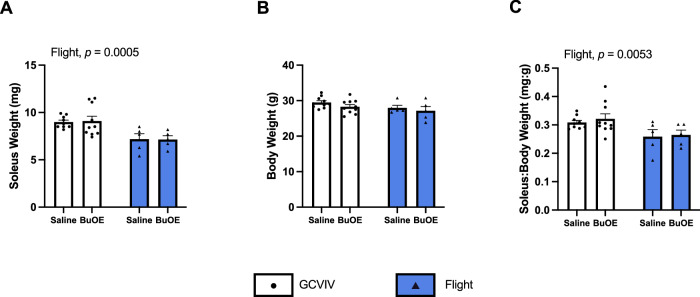
Spaceflight induces soleus muscle atrophy that is not attenuated by BuOE treatment. Soleus weight is significantly reduced following spaceflight (**A**) with no changes in body weight (**B**), resulting in a significant reduction of the soleus:body weight ratio in the flight groups compared to controls (**C**). All values are mean ± SEM with *p*-values presented above the graphs.

Ionophore supported Ca^2+^-dependent SERCA ATPase activity was assessed across a range of [Ca^2+^] (*p*Ca 7.0–5.0) and was presented as percentage of maximal activity (Fig. 2A) to assess the *p*Ca_50_ (i.e., [Ca^2+^] required to elicit ½ maximal activity) as a measure of SERCA’s apparent affinity for Ca^2+^. There was no effect of spaceflight or BuOE treatment on maximal SERCA ATPase activity (Fig. 2B), but a main effect of BuOE treatment increasing SERCA’s apparent affinity for Ca^2+^ was detected (*p* = 0.0323, Fig. 2C). In dividing the maximal ATPase rates with ionophore (no Ca^2+^ gradient) by the rates without ionophore (Ca^2+^ gradient), a measure of SR Ca^2+^ permeability and leak can be obtained^34–36^. In doing this, we observed a main effect of flight reducing the ionophore ratio (*p* = 0.0255, Fig. 2D), indicative of increased SR Ca^2+^ leak.

**Fig. 2:**
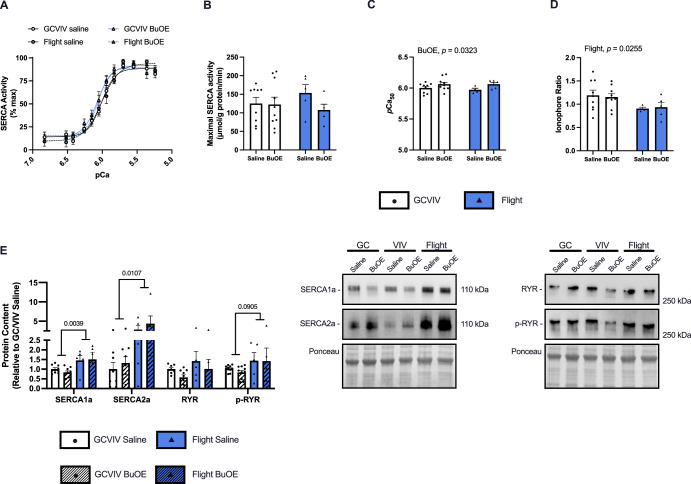
BuOE increases SERCA’s affinity for Ca^2+^ and exposure to microgravity reduces SERCA’s ionophore ratio. SERCA activity-*p*Ca curves presented as % max (**A**). No changes in maximal SERCA ATPase activity were detected (**B**); but, a main effect of BuOE increasing the *p*Ca_50_ was found (**C**). The ionophore ratio was significantly reduced in spaceflight groups with no effect of BuOE (**D**). Densitometric analysis and representative western blot images show significant increases in SERCA1a and SERCA2a protein content in the space-flown soleus (**E**). All values are mean ± SEM with *p*-values presented above the graphs (**A**–**D**) or above bars (**E**).

Western blotting revealed significant increases in SERCA1a (*p* = 0.0039) and SERCA2a (*p* = 0.0107) protein content in the spaceflight groups compared to GC/VIV with no effect of BuOE treatment (Fig. 2E). Total and phosphorylated content of the Ca^2+^ release protein, RYR, was also investigated with no effects on total RYR, but increases in p-RYR (increases Ca^2+^ release) in flight groups compared to GC/VIV, though this did not reach statistical significance (*p* = 0.0905, Fig. 2E).

4-HNE is a product of lipid peroxidation due to increased RONS and therefore serves as a marker of oxidative stress^37^. Significant increases in 4-HNE were observed in flight groups compared to GC/VIV (*p* = 0.0387) with no effect of BuOE treatment (Fig. 3A). Further, total SOD content was reduced in flight groups compared to controls, though this did not reach statistical significance (*p* = 0.0510, Fig. 3A). Investigating protein content of the three SERCA regulators demonstrated dynamic changes in response to spaceflight. Significant reductions in SLN (*p* = 0.0080), increases in NNAT (*p* = 0.0158), and reductions in PLN approaching statistical significance (*p* = 0.0828) were all observed in the space-flown soleus compared to GC/VIV (Fig. 3B).

**Fig. 3:**
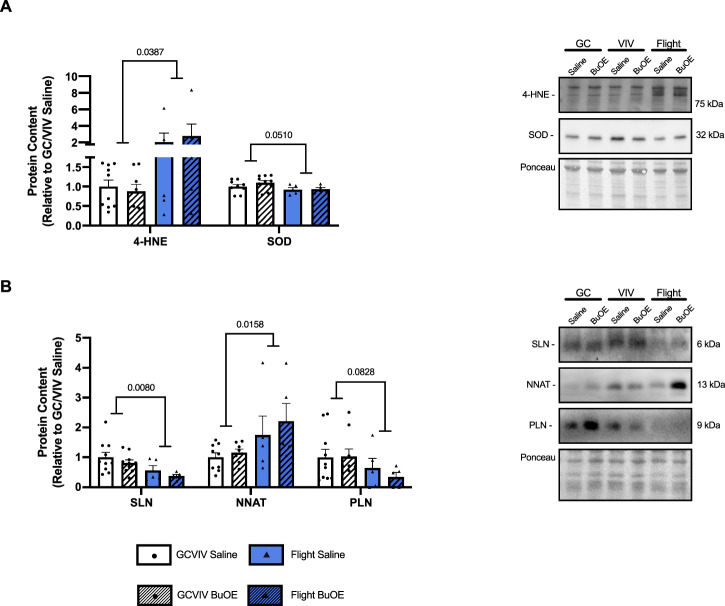
BuOE treatment does not attenuate increased oxidative stress following spaceflight. Densitometric analysis and representative western blots show significant increases in 4-HNE following spaceflight and reductions in SOD approaching statistical significance with no effects of BuOE treatment (**A**). Protein content of the three SERCA regulators shows significant reductions in SLN and PLN and significant increases in NNAT following spaceflight (**B**). All values are mean ± SEM with *p*-values presented in bars.

To extend our previous work demonstrating that soleus SERCA Ca^2+^ uptake is impaired following spaceflight, which we attributed to increased RONS content^5^, we investigated whether treatment with the antioxidant, BuOE, could attenuate muscle atrophy and SERCA dysfunction following spaceflight. We found BuOE was unable to attenuate soleus muscle atrophy following 35 days in space. While BuOE treatment increased SERCA’s affinity for Ca^2+^ in both the GC/VIV and flight groups, it was unable to rescue the significant increases in 4-HNE content or SR Ca^2+^ leak, measured using an ionophore ratio, induced by microgravity exposure. Consistent with previous work^5^, we observed increases in protein content of both SERCA isoforms following spaceflight as well as increases in oxidative stress and dynamic changes in the expression of the three SERCA regulators, SLN, NNAT, and PLN. Spaceflight also tended to reduce SOD content and increase p-RYR, both of which may be contributing to increased SR Ca^2+^ leak.

Past literature using both ground-based and spaceflight models in rodents and humans has begun to suggest that Ca^2+^ dysregulation in skeletal muscle may be an early step in the pathogenesis of weakness and atrophy^4,5,38^. Specifically, we have previously shown the Ca^2+^ uptake ability of SERCA to be impaired following spaceflight which we attributed, at least partially, to increases in total protein RONS^5^. SERCA pumps have highly susceptible tyrosine residues that, in the presence of increased RONS, can result in tyrosine nitration^15–18^. Tyrosine 294 and 295 of SERCA appear to be most vulnerable to tyrosine nitration and reside in the transmembrane domain of the pump, a necessary domain for Ca^2+^ translocation^16^. The structural changes that ensue following tyrosine nitration of these residues result in impaired catalytic activity and increased [Ca^2+^]_i_. Here, we investigated ATPase activity of the SERCA pump and found no changes in response to microgravity; though, we do observe a main effect of BuOE treatment increasing SERCA’s affinity for Ca^2+^. This is consistent with our previous work showing that heterozygous deletion of SOD2 reduces SERCA’s affinity for Ca^2+^^20^. The increases in both SERCA isoforms with spaceflight are consistent with our previous work^5^ and were similarly unable to attenuate spaceflight-induced increases in [Ca^2+^]_i_. Given that SERCA density is the primary determinant of Ca^2+^ uptake^39^ the increased protein content should increase SERCA catalytic activity; however, this was not detected, suggesting protein dysfunction or a failed compensatory response. The mechanism underlying increased SERCA protein expression following spaceflight requires further investigation but has been observed in other muscle-wasting diseases and myopathies^21,40,41^. SLN and PLN act to reduce SERCA-mediated Ca^2+^ uptake through reductions in affinity as well as inducing Ca^2+^ slippage^42–44^; however, no effects of BuOE were detected on the protein expression of SLN, NNAT, or PLN with the only changes being observed with spaceflight. With regards to the SERCA regulators, inconsistencies were observed with the previous RR-9 mission where there was a clear increase in SLN and decrease in NNAT following spaceflight^5^; but, RR-18 shows the opposite with reductions in SLN and increases in NNAT, which is maintained even when loaded on the same gel (Supplemental Fig. 1A, B). At present, we can only speculate that possible differences in reloading time (~20 h for RR-18^45^ and ~38 h for RR-9^46^) would lead to altered expression patterns, but this warrants further investigation.

It has been well established that Ca^2+^ accumulates in the cytosol of muscle fibers following unloading^7,47–51^ and we also contribute to the growing literature on Ca^2+^ dysregulation by demonstrating that SR Ca^2+^ leak is significantly increased in the murine soleus following microgravity exposure, consistent with what has been reported with hindlimb suspension^52^. Increased SR Ca^2+^ leak has been observed in various muscle pathologies, many of which are related to post-translational modifications of RYR and its regulators^53–56^. Here, we observed increases in phosphorylated RYR, albeit non-statistically significant, that may still be contributing to increased Ca^2+^ leak. Notably, previous work has demonstrated that blocking L-type Ca^2+^ channels attenuates unloading-induced Ca^2+^ overload in the rat soleus^57^. In addition to alterations to RYR, the lipid composition of the SR membrane can also drastically alter Ca^2+^ handling^34,58,59^. More specifically, it has been shown that increased lipid peroxidation will increase membrane permeability^60–62^ and with significant increases in 4-HNE, a product of lipid peroxidation^37^, in the space-flown soleus, it is likely that alterations to SR permeability are contributing to increased Ca^2+^ leak, though more targeted studies are required. As well as SR Ca^2+^ leak, there are several other mechanisms that can lead to elevations in [Ca^2+^]_i_ that were not explored in the present study. One such mechanism is early muscle membrane depolarization, due to reduced Na^+^/K^+^-ATPase pump activity^63,64^, possibly triggering the opening of the voltage-sensitive dihydropyridine receptors and increasing [Ca^2+^]_i_. Ca^2+^ overload may also be a product of store-operated Ca^2+^ entry - the process of Ca^2+^ influx triggered by reductions in SR luminal [Ca^2+^] (for review, see ref. ^65^), which could be triggered by SERCA dysfunction. This phenomenon has been identified to contribute to Ca^2+^ overload in dystrophic muscle^66,67^ and various other myopathies^68^, though its contributions to muscle atrophy and weakness following unloading remain to be explored.

In skeletal muscle, BuOE was unable to attenuate the increases in 4-HNE caused by spaceflight; but, was effective in reducing 4-HNE in the retina from the same mice^45^, indicative of tissue-specific responses to BuOE at the dose provided in this study. Though speculative, it is possible that BuOE was unable to compensate for the reductions in SOD content observed following spaceflight, an effect also seen in astronaut muscle following spaceflight, albeit in hair samples^69^. Future studies focused on skeletal muscle, namely SERCA function in the soleus, may benefit from higher doses of BuOE treatment or other modes of cytotoxic protection. For example, HSP70 can protect SERCA from oxidant damage in conditions of muscular dystrophy^70^, and it would be of interest to determine whether pharmacological induction or genetic overexpression of HSP70 could protect SERCA function in the context of spaceflight. While we were limited in our ability to measure the sources of RONS in this study, previous work has demonstrated mitochondrial dysfunction^71,72^, and increased NADPH oxidase expression and activity^73,74^ to contribute to RONS production with spaceflight and simulated microgravity exposure. Further investigation into whether targeting RONS production at the source can counteract muscle weakness and atrophy is needed. Regardless, targeting Ca^2+^ leak from the SR has shown to be beneficial to muscle health in aged mice^75^, hypoxia-induced atrophy in rodents^76^, and murine dystrophic muscle^77^ indicating that this may be a viable target for spaceflight-induced changes in SR permeability and leak. Importantly, a recent study by Sharlo, et al. ^78^ treated rats with the SERCA activator, CDN1163, during hindlimb suspension (NASA’s simulated model of microgravity) and, while they didn’t measure SERCA function directly, they found improvements in several muscle parameters including soleus fatigue resistance, mitochondrial markers, and markers of Ca^2+^ homeostasis. This finding, along with others targeting SERCA activation in situations of muscle impairment^11,12^ demonstrate the importance of directly targeting SERCA in space.

We observe some discrepancies related to SLN expression following spaceflight between the RR-9^5^ and the present RR-18 study. SLN expression has been shown to upregulated in several muscle-wasting conditions, but whether this upregulation is beneficial or detrimental to muscle is still under debate (for review, see ref. ^79^). Procedurally, the RR-1, RR-9, and RR-18 studies all maintained mouse strain and housing temperature but disparities in reloading time prior to euthanasia (RR-9, ~38 h^46^; RR-18, ~20 h^45^) may contribute to the differences in SLN expression between missions. This hypothesis remains speculative but would be an interesting avenue of investigation in the future. Finally, due to limitations on the amount of sample, we were unable to investigate SERCA-specific analysis of RONS modifications which could provide insight into the changes in Ca^2+^ affinity observed here.

In summary, with soleus muscles from space-flown male mice, we investigated the effects of antioxidant treatment with BuOE on soleus muscle atrophy, SERCA ATPase function, and SR Ca^2+^ leak. We found spaceflight to induce soleus muscle atrophy and increases in SR Ca^2+^ leak that could not be overcome by BuOE treatment, despite increases in SERCA’s affinity for Ca^2+^. Future studies aimed at reducing Ca^2+^ leak or increasing SERCA activation could reveal SERCA and Ca^2+^ handling as viable targets to counteract weakness and atrophy observed with spaceflight.

## Methods

### Muscle samples

Soleus muscles were obtained from the NASA Biological Institutional Scientific Collection. Muscles came from male C57BL/6 J mice from the RR-18 mission which had two control groups, ground control (GC) and vivarium (VIV) control, as well as a flight group which had mice housed on the International Space Station for 35 days as previously described^5,45,80–83^. All mice were treated weekly with either BuOE (1 mg/kg) or saline via subcutaneous injection beginning one week prior to launch and continuing for the duration of spaceflight. Upon live return to Earth, soleus muscles were dissected and stored in RNALater at −80 °C. Muscles were thawed, rinsed from RNALater, and homogenized in homogenizing buffer prior to further analysis^5^.

### SERCA activity assay and ionophore ratio

SERCA ATPase activity was assessed using an enzyme-linked spectrophotometric assay as previously described^19^. Full SERCA ATPase-*p*Ca curves were run in the presence of ionophore as well as in the absence of ionophore specifically at maximal stimulating Ca^2+^ concentrations (*p*Ca = 5.0) to gain a measure of SR Ca^2+^ leak and permeability as previously described^34–36^. The Ca^2+^ ionophore, A23187 induces SR membrane permeability, which encourages maximal SERCA activity by preventing back inhibition. Thus, if the SR membrane is inherently ‘leaky’ then the calculated ionophore ratio (maximal SERCA activity in the presence of ionophore: maximal SERCA activity in the absence of ionophore) will be relatively smaller.

### Western blotting

Western blotting was performed on muscle homogenate to assess protein content for SERCA1a, SERCA2a, total and phosphorylated content of the Ca^2+^ release protein ryanodine receptor (RYR1/2), 4-hydroxynonenal (4-HNE) as a marker of oxidative stress, SOD, and the three SERCA regulators, sarcolipin (SLN), neuronatin (NNAT), and phospholamban (PLN) as previously described^5,19^. Specific methods for the aforementioned proteins are listed in Supplemental Table 1.

### Statistical analysis

All data are presented as means ± standard error of the mean (SEM). No statistical differences were detected between the GC and VIV groups and so they were combined within their respective treatment groups to increase statistical power. A two-way ANOVA with Tukey’s post hoc test was used to compare the main effects of GC/VIV vs flight and saline vs BuOE as well as any interaction that may exist. Statistical significance was set at *p* ≤ 0.05 and outliers were detected and removed prior to analysis if they were ±2 standard deviations from the mean of their respective group. All statistical tests were employed using GraphPad Prism 9. *p*-values are presented above graphs.

### Supplementary information

Supplementary Information

## Data Availability

The data that support this study are available at the NASA Open Science Data Repository (10.26030/mx0n-ta73).

## References

[1] Sandona, D. et al. Adaptation of mouse skeletal muscle to long-term microgravity in the MDS mission. *PLoS ONE* **7**, e33232 (2012). 22470446 10.1371/journal.pone.0033232 PMC3314659

[2] Okada, R. et al. Transcriptome analysis of gravitational effects on mouse skeletal muscles under microgravity and artificial 1 g onboard environment. *Sci. Rep.* **11**, 9168 (2021). 33911096 10.1038/s41598-021-88392-4 PMC8080648

[3] Widrick, J. J. et al. Effect of a 17 day spaceflight on contractile properties of human soleus muscle fibres. *J. Physiol.* **516**, 915–930 (1999). 10200437 10.1111/j.1469-7793.1999.0915u.x PMC2269300

[4] Monti, E. et al. Neuromuscular junction instability and altered intracellular calcium handling as early determinants of force loss during unloading in humans. *J. Physiol.* **599**, 3037–3061 (2021). 33881176 10.1113/JP281365 PMC8359852

[5] Braun, J. L., Geromella, M. S., Hamstra, S. I., Messner, H. N. & Fajardo, V. A. Characterizing SERCA function in murine skeletal muscles after 35–37 days of spaceflight. *Int. J. Mol. Sci.*10.3390/ijms222111764 (2021). 10.3390/ijms222111764 PMC8584217 34769190

[6] Baranowski, R. W. et al. Toward countering muscle and bone loss with spaceflight: GSK3 as a potential target. *iScience* **26**, 107047 (2023). 37360691 10.1016/j.isci.2023.107047 PMC10285634

[7] Ingalls, C. P., Wenke, J. C. & Armstrong, R. B. Time course changes in [Ca2+]i, force, and protein content in hindlimb-suspended mouse soleus muscles. *Aviat. Space Environ. Med.* **72**, 471–476 (2001). 11346014

[8] Periasamy, M. & Huke, S. SERCA pump level is a critical determinant of Ca(2+)homeostasis and cardiac contractility. *J. Mol. Cell. Cardiol.* **33**, 1053–1063 (2001). 11444913 10.1006/jmcc.2001.1366

[9] Misquitta, C. M., Mack, D. P. & Grover, A. K. Sarco/endoplasmic reticulum Ca2+ (SERCA)-pumps: link to heart beats and calcium waves. *Cell Calcium* **25**, 277–290 (1999). 10456225 10.1054/ceca.1999.0032

[10] Eisner, V., Csordás, G. & Hajnóczky, G. Interactions between sarco-endoplasmic reticulum and mitochondria in cardiac and skeletal muscle - pivotal roles in Ca²^+^ and reactive oxygen species signaling. *J. Cell Sci.* **126**, 2965–2978 (2013). 23843617 10.1242/jcs.093609 PMC3711195

[11] Qaisar, R. et al. Restoration of SERCA ATPase prevents oxidative stress-related muscle atrophy and weakness. *Redox Biol.* **20**, 68–74 (2019). 30296699 10.1016/j.redox.2018.09.018 PMC6174848

[12] Qaisar, R. et al. Restoration of sarcoplasmic reticulum Ca(2+) ATPase (SERCA) activity prevents age-related muscle atrophy and weakness in mice. *Int. J. Mol. Sci.*10.3390/ijms22010037 (2020). 10.3390/ijms22010037 PMC7792969 33375170

[13] Enns, D. L. & Belcastro, A. N. Early activation and redistribution of calpain activity in skeletal muscle during hindlimb unweighting and reweighting. *Can. J. Physiol. Pharm.* **84**, 601–609 (2006). 10.1139/y06-013 16900244

[14] Hosfield, C. M., Elce, J. S., Davies, P. L. & Jia, Z. Crystal structure of calpain reveals the structural basis for Ca(2+)-dependent protease activity and a novel mode of enzyme activation. *EMBO J.* **18**, 6880–6889 (1999). 10601010 10.1093/emboj/18.24.6880 PMC1171751

[15] Viner, R. I., Ferrington, D. A., Huhmer, A. F., Bigelow, D. J. & Schoneich, C. Accumulation of nitrotyrosine on the SERCA2a isoform of SR Ca-ATPase of rat skeletal muscle during aging: a peroxynitrite-mediated process? *FEBS Lett.* **379**, 286–290 (1996). 8603707 10.1016/0014-5793(95)01530-2

[16] Viner, R. I., Ferrington, D. A., Williams, T. D., Bigelow, D. J. & Schöneich, C. Protein modification during biological aging: selective tyrosine nitration of the SERCA2a isoform of the sarcoplasmic reticulum Ca2+-ATPase in skeletal muscle. *Biochem. J.* **340**, 657–669 (1999). 10359649 10.1042/bj3400657 PMC1220296

[17] Viner, R. I., Williams, T. D. & Schöneich, C. Peroxynitrite modification of protein thiols: oxidation, nitrosylation, and S-glutathiolation of functionally important cysteine residue(s) in the sarcoplasmic reticulum Ca-ATPase. *Biochemistry* **38**, 12408–12415 (1999). 10493809 10.1021/bi9909445

[18] Tupling, A. R. et al. Effects of buthionine sulfoximine treatment on diaphragm contractility and SR Ca2+ pump function in rats. *J. Appl. Physiol.* **103**, 1921–1928 (2007). 17717121 10.1152/japplphysiol.00529.2007

[19] Braun, J. L., Hamstra, S. I., Messner, H. N. & Fajardo, V. A. SERCA2a tyrosine nitration coincides with impairments in maximal SERCA activity in left ventricles from tafazzin-deficient mice. *Physiol. Rep.* **7**, e14215 (2019). 31444868 10.14814/phy2.14215 PMC6708055

[20] Braun, J. L. et al. Heterozygous SOD2 deletion selectively impairs SERCA function in the soleus of female mice. *Physiol. Rep.* **10**, e15285 (2022). 35581738 10.14814/phy2.15285 PMC9114654

[21] Cleverdon, R. E. G. et al. Sarco(endo)plasmic reticulum Ca(2+)-ATPase function is impaired in skeletal and cardiac muscles from young DBA/2J mdx mice. *iScience* **25**, 104972 (2022). 36093052 10.1016/j.isci.2022.104972 PMC9459692

[22] Qaisar, R. et al. Oxidative stress-induced dysregulation of excitation-contraction coupling contributes to muscle weakness. *J. Cachexia Sarcopenia Muscle* **9**, 1003–1017 (2018). 30073804 10.1002/jcsm.12339 PMC6204588

[23] Fink, B. D., Bai, F., Yu, L. & Sivitz, W. I. Regulation of ATP production: dependence on calcium concentration and respiratory state. *Am. J. Physiol. Cell Physiol.* **313**, C146–c153 (2017). 28515085 10.1152/ajpcell.00086.2017

[24] Glancy, B., Willis, W. T., Chess, D. J. & Balaban, R. S. Effect of calcium on the oxidative phosphorylation cascade in skeletal muscle mitochondria. *Biochemistry* **52**, 2793–2809 (2013). 23547908 10.1021/bi3015983 PMC4157357

[25] Kavanagh, N. I., Ainscow, E. K. & Brand, M. D. Calcium regulation of oxidative phosphorylation in rat skeletal muscle mitochondria. *Biochim. Biophys. Acta* **1457**, 57–70 (2000). 10692550 10.1016/S0005-2728(00)00054-2

[26] Pacher, P., Beckman, J. S. & Liaudet, L. Nitric oxide and peroxynitrite in health and disease. *Physiol. Rev.* **87**, 315–424 (2007). 17237348 10.1152/physrev.00029.2006 PMC2248324

[27] Miao, L. & St Clair, D. K. Regulation of superoxide dismutase genes: implications in disease. *Free Radic. Biol. Med.* **47**, 344–356 (2009). 19477268 10.1016/j.freeradbiomed.2009.05.018 PMC2731574

[28] Batinic-Haberle, I., Tovmasyan, A. & Spasojevic, I. An educational overview of the chemistry, biochemistry and therapeutic aspects of Mn porphyrins–from superoxide dismutation to H2O2-driven pathways. *Redox Biol.* **5**, 43–65 (2015). 25827425 10.1016/j.redox.2015.01.017 PMC4392060

[29] Batinic-Haberle, I. et al. SOD therapeutics: latest insights into their structure-activity relationships and impact on the cellular redox-based signaling pathways. *Antioxid. Redox Signal.* **20**, 2372–2415 (2014). 23875805 10.1089/ars.2012.5147 PMC4005498

[30] Vujaskovic, Z. et al. A small molecular weight catalytic metalloporphyrin antioxidant with superoxide dismutase (SOD) mimetic properties protects lungs from radiation-induced injury. *Free Radic. Biol. Med.* **33**, 857–863 (2002). 12208373 10.1016/S0891-5849(02)00980-2

[31] Pearlstein, R. D. et al. Metalloporphyrin antioxidants ameliorate normal tissue radiation damage in rat brain. *Int. J. Radiat. Biol.* **86**, 145–163 (2010). 20148700 10.3109/09553000903419965

[32] Mao, X. W. et al. Radioprotective effect of a metalloporphyrin compound in rat eye model. *Curr. Eye Res.* **34**, 62–72 (2009). 19172472 10.1080/02713680802546948

[33] Mao, X. W., Crapo, J. D. & Gridley, D. S. Mitochondrial oxidative stress-induced apoptosis and radioprotection in proton-irradiated rat retina. *Radiat. Res.* **178**, 118–125 (2012). 22780102 10.1667/RR2821.1

[34] Fajardo, V. A. et al. Dietary docosahexaenoic acid supplementation reduces SERCA Ca2+ transport efficiency in rat skeletal muscle. *Chem. Phys. Lipids* **187**, 56–61 (2015). 25772907 10.1016/j.chemphyslip.2015.03.001

[35] Bombardier, E., Smith, I. C., Vigna, C., Fajardo, V. A. & Tupling, A. R. Ablation of sarcolipin decreases the energy requirements for Ca2+ transport by sarco(endo)plasmic reticulum Ca2+-ATPases in resting skeletal muscle. *FEBS Lett.* **587**, 1687–1692 (2013). 23628781 10.1016/j.febslet.2013.04.019

[36] Tupling, R., Green, H. & Tupling, S. Partial ischemia reduces the efficiency of sarcoplasmic reticulum Ca2+ transport in rat EDL. *Mol. Cell. Biochem.* **224**, 91–102 (2001). 11693204 10.1023/A:1011930502758

[37] Yin, H., Xu, L. & Porter, N. A. Free radical lipid peroxidation: mechanisms and analysis. *Chem. Rev.* **111**, 5944–5972 (2011). 21861450 10.1021/cr200084z

[38] Altaeva, E. G., Ogneva, I. V. & Shenkman, B. Dynamics of calcium ions accumulation and changes of isoforms of sarcoplasmic reticulum Ca-ATPase (SERCA) in soleus muscle fibers of rats and Mongolian gerbils under gravitational unloading of various duration. *Cell Tissue Biol.* **4**, 594–599 (2010). 10.1134/S1990519X10060118

[39] Tupling, A. R. The sarcoplasmic reticulum in muscle fatigue and disease: role of the Sarco (endo)plasmic reticulum Ca2+-ATPase. *Can. J. Appl. Physiol.* **29**, 308–329 (2004). 15199229 10.1139/h04-021

[40] Kanazawa, Y., Takahashi, T., Nagano, M., Koinuma, S. & Shigeyoshi, Y. The effects of aging on sarcoplasmic reticulum-related factors in the skeletal muscle of mice. *Int. J. Mol. Sci.*10.3390/ijms25042148 (2024). 10.3390/ijms25042148 PMC10889371 38396828

[41] Fajardo, V. A. et al. Phospholamban overexpression in mice causes a centronuclear myopathy-like phenotype. *Dis. Model. Mech.* **8**, 999–1009 (2015). 26035394 10.1242/dmm.020859 PMC4527296

[42] Braun, J. L. et al. Neuronatin promotes SERCA uncoupling and its expression is altered in skeletal muscles of high-fat diet-fed mice. *FEBS Lett.* **595**, 2756–2767 (2021). 34693525 10.1002/1873-3468.14213

[43] Stammers, A. N. et al. The regulation of sarco(endo)plasmic reticulum calcium-ATPases (SERCA). *Can. J. Physiol. Pharmacol.* **93**, 843–854 (2015). 25730320 10.1139/cjpp-2014-0463

[44] Smith, W. S., Broadbridge, R., East, J. M. & Lee, A. G. Sarcolipin uncouples hydrolysis of ATP from accumulation of Ca2+ by the Ca2+-ATPase of skeletal-muscle sarcoplasmic reticulum. *Biochem. J.* **361**, 277–286 (2002). 11772399 10.1042/bj3610277 PMC1222307

[45] Mao, X., Stanbouly, S., Holley, J., Pecaut, M. & Crapo, J. Evidence of spaceflight-induced adverse effects on photoreceptors and retinal function in the mouse eye. *Int. J. Mol. Sci.*10.3390/ijms24087362 (2023). 10.3390/ijms24087362 PMC10138634 37108526

[46] Mao, X. W. et al. Characterization of mouse ocular response to a 35-day spaceflight mission: evidence of blood-retinal barrier disruption and ocular adaptations. *Sci. Rep.* **9**, 8215 (2019). 31160660 10.1038/s41598-019-44696-0 PMC6547757

[47] Arutyunyan, R. et al. The contraction of unweighted fast and slow rat muscles in calcium-free solution. *Basic Appl. Myol.* **5**, 169–175 (1995).

[48] Ingalls, C. P., Warren, G. L. & Armstrong, R. B. Intracellular Ca2+ transients in mouse soleus muscle after hindlimb unloading and reloading. *J. Appl. Physiol.* **87**, 386–390 (1999). 10409599 10.1152/jappl.1999.87.1.386

[49] Nemirovskaya, T. L. & Sharlo, K. A. Roles of ATP and SERCA in the regulation of calcium turnover in unloaded skeletal muscles: current view and future directions. *Int. J. Mol. Sci.*10.3390/ijms23136937 (2022). 10.3390/ijms23136937 PMC9267070 35805949

[50] Shenkman, B. S. How postural muscle senses disuse? Early signs and signals. *Int. J. Mol. Sci.*10.3390/ijms21145037 (2020). 10.3390/ijms21145037 PMC7404025 32708817

[51] Shenkman, B. S. & Nemirovskaya, T. L. Calcium-dependent signaling mechanisms and soleus fiber remodeling under gravitational unloading. *J. Muscle Res. Cell Motil.* **29**, 221–230 (2008). 19130271 10.1007/s10974-008-9164-7

[52] Yoshioka, T., Shirota, T., Tazoe, T. & Yamashita-Goto, K. Calcium movement of sarcoplasmic reticulum from hindlimb suspended muscle. *Acta Astronaut.* **38**, 209–212 (1996). 11540780 10.1016/0094-5765(96)00010-0

[53] Kushnir, A. et al. Intracellular calcium leak as a therapeutic target for RYR1-related myopathies. *Acta Neuropathol.* **139**, 1089–1104 (2020). 32236737 10.1007/s00401-020-02150-w PMC7788518

[54] Lotteau, S. et al. A mechanism for statin-induced susceptibility to myopathy. *JACC Basic Transl. Sci.* **4**, 509–523 (2019). 31468006 10.1016/j.jacbts.2019.03.012 PMC6712048

[55] Kushnir, A. et al. Ryanodine receptor calcium leak in circulating B-lymphocytes as a biomarker in heart failure. *Circulation* **138**, 1144–1154 (2018). 29593014 10.1161/CIRCULATIONAHA.117.032703 PMC6162180

[56] Robin, G., Berthier, C. & Allard, B. Sarcoplasmic reticulum Ca2+ permeation explored from the lumen side in mdx muscle fibers under voltage control. *J. Gen. Physiol.* **139**, 209–218 (2012). 22371362 10.1085/jgp.201110738 PMC3289961

[57] Mukhina, A. M., Altaeva, E. G., Nemirovskaya, T. L. & Shenkman, B. S. The role of L-type calcium channels in the accumulation of Ca2+ in soleus muscle fibers in the rat and changes in the ratio of myosin and serca isoforms in conditions of gravitational unloading. *Neurosci. Behav. Physiol.* **38**, 181–188 (2008). 18197386 10.1007/s11055-008-0027-x

[58] Froud, R. J., Earl, C. R., East, J. M. & Lee, A. G. Effects of lipid fatty acyl chain structure on the activity of the (Ca2+ + Mg2+)-ATPase. *Biochim. Biophys. Acta* **860**, 354–360 (1986). 2943317 10.1016/0005-2736(86)90532-8

[59] Cornea, R. L. & Thomas, D. D. Effects of membrane thickness on the molecular dynamics and enzymatic activity of reconstituted Ca-ATPase. *Biochemistry* **33**, 2912–2920 (1994). 8130205 10.1021/bi00176a022

[60] Van der Paal, J., Neyts, E. C., Verlackt, C. C. W. & Bogaerts, A. Effect of lipid peroxidation on membrane permeability of cancer and normal cells subjected to oxidative stress. *Chem. Sci.* **7**, 489–498 (2016). 28791102 10.1039/C5SC02311D PMC5518669

[61] Hirata, Y. et al. Lipid peroxidation increases membrane tension, Piezo1 gating, and cation permeability to execute ferroptosis. *Curr. Biol.* **33**, 1282–1294.e1285 (2023). 36898371 10.1016/j.cub.2023.02.060

[62] Wong-Ekkabut, J. et al. Effect of lipid peroxidation on the properties of lipid bilayers: a molecular dynamics study. *Biophys. J.* **93**, 4225–4236 (2007). 17766354 10.1529/biophysj.107.112565 PMC2098729

[63] Kravtsova, V. V. et al. Isoform-specific Na,K-ATPase alterations precede disuse-induced atrophy of rat soleus muscle. *Biomed. Res. Int.* **2015**, 720172 (2015). 25654120 10.1155/2015/720172 PMC4309216

[64] Kravtsova, V. V. et al. Chronic ouabain prevents Na,K-ATPase dysfunction and targets AMPK and IL-6 in disused rat soleus muscle. *Int. J. Mol. Sci.*10.3390/ijms22083920 (2021). 10.3390/ijms22083920 PMC8069997 33920198

[65] Avila-Medina, J. et al. In *Calcium Signaling* (ed. Islam, M. S.) 489–504 (Springer Int. Publishing, 2020).

[66] Edwards, J. N. et al. Upregulation of store-operated Ca2+ entry in dystrophic mdx mouse muscle. *Am. J. Physiol. Cell Physiol.* **299**, C42–C50 (2010). 20427714 10.1152/ajpcell.00524.2009

[67] Vandebrouck, C., Martin, D., Colson-Van Schoor, M., Debaix, H. & Gailly, P. Involvement of TRPC in the abnormal calcium influx observed in dystrophic (mdx) mouse skeletal muscle fibers. *J. Cell Biol.* **158**, 1089–1096 (2002). 12235126 10.1083/jcb.200203091 PMC2173225

[68] Lacruz, R. S. & Feske, S. Diseases caused by mutations in ORAI1 and STIM1. *Ann. N. Y. Acad. Sci.* **1356**, 45–79 (2015). 26469693 10.1111/nyas.12938 PMC4692058

[69] Indo, H. P. et al. Changes in mitochondrial homeostasis and redox status in astronauts following long stays in space. *Sci. Rep.* **6**, 39015 (2016). 27982062 10.1038/srep39015 PMC5159838

[70] Gehrig, S. M. et al. Hsp72 preserves muscle function and slows progression of severe muscular dystrophy. *Nature* **484**, 394–398 (2012). 22495301 10.1038/nature10980

[71] da Silveira, W. A. et al. Comprehensive multi-omics analysis reveals mitochondrial stress as a central biological hub for spaceflight impact. *Cell* **183**, 1185–1201. e1120 (2020). 33242417 10.1016/j.cell.2020.11.002 PMC7870178

[72] Garrett-Bakelman, F. E. et al. The NASA Twins Study: a multidimensional analysis of a year-long human spaceflight. *Science*10.1126/science.aau8650 (2019). 10.1126/science.aau8650 PMC7580864 30975860

[73] Mao, X. W. et al. Role of NADPH oxidase as a mediator of oxidative damage in low-dose irradiated and hindlimb-unloaded mice. *Radiat. Res.* **188**, 392–399 (2017). 28763287 10.1667/RR14754.1

[74] Kumar, A., Tahimic, C. G., Almeida, E. A. & Globus, R. K. Spaceflight modulates the expression of key oxidative stress and cell cycle related genes in heart. *Int. J. Mol. Sci.* **22**, 9088 (2021). 34445793 10.3390/ijms22169088 PMC8396460

[75] Andersson, D. C. et al. Ryanodine receptor oxidation causes intracellular calcium leak and muscle weakness in aging. *Cell Metab.* **14**, 196–207 (2011). 21803290 10.1016/j.cmet.2011.05.014 PMC3690519

[76] Agrawal, A. et al. Redox modification of ryanodine receptor contributes to impaired Ca(2+) homeostasis and exacerbates muscle atrophy under high altitude. *Free Radic. Biol. Med.* **160**, 643–656 (2020). 32916280 10.1016/j.freeradbiomed.2020.09.001

[77] Bellinger, A. M. et al. Hypernitrosylated ryanodine receptor calcium release channels are leaky in dystrophic muscle. *Nat. Med.* **15**, 325–330 (2009). 19198614 10.1038/nm.1916 PMC2910579

[78] Sharlo, K. A. et al. The effect of SERCA activation on functional characteristics and signaling of rat soleus muscle upon 7 days of unloading. *Biomolecules*10.3390/biom13091354 (2023). 10.3390/biom13091354 PMC10526198 37759754

[79] Chambers, P. J., Juracic, E. S., Fajardo, V. A. & Tupling, A. R. Role of SERCA and sarcolipin in adaptive muscle remodeling. *Am. J. Physiol. Cell Physiol.* **322**, C382–c394 (2022). 35044855 10.1152/ajpcell.00198.2021

[80] Choi, S. Y. et al. Validation of a new rodent experimental system to investigate consequences of long duration space habitation. *Sci. Rep.* **10**, 2336 (2020). 32047211 10.1038/s41598-020-58898-4 PMC7012842

[81] Sun, G. S. et al. Evaluation of the nutrient-upgraded rodent food bar for rodent spaceflight experiments. *Nutrition* **26**, 1163–1169 (2010). 20116210 10.1016/j.nut.2009.09.018

[82] Overbey, E. G. et al. Spaceflight influences gene expression, photoreceptor integrity, and oxidative stress-related damage in the murine retina. *Sci. Rep.* **9**, 13304 (2019). 31527661 10.1038/s41598-019-49453-x PMC6746706

[83] Kremsky, I. et al. Spaceflight-induced gene expression profiles in the mouse brain are attenuated by treatment with the antioxidant BuOE. *Int. J. Mol. Sci.*10.3390/ijms241713569 (2023). 10.3390/ijms241713569 PMC10487739 37686374

