# Supplementary material for: Spaceflight increases sarcoplasmic reticulum Ca2+ leak and this cannot be counteracted with BuOE treatment
Source: NPJ Microgravity. 2024 Jul 19;10:78. doi: 10.1038/s41526-024-00419-y (PMC11271499; doi:10.1038/s41526-024-00419-y)
Supplement: Supplementary file 1 — Supplementary Information [file 41526_2024_419_MOESM1_ESM.pdf]

Spaceflight increases sarcoplasmic reticulum Ca^{2+} leak and this cannot be counteracted with BuOE treatment

Jessica L. Braun[1,2] & Val A. Fajardo[1,2*]

[1]Department of Kinesiology, Brock University, St. Catharines, ON, Canada

[2]Centre for Bone and Muscle Health, Brock University, St. Catharines, ON, Canada

SUPPLEMENTARY MATERIAL

Supplemental Table 1. Protein specific Western blotting protocols and materials.

Target	Protein Loaded (μg)	Type of Gel	Membrane	Primary Antibody Dilution	Primary Antibody Details
**SERCA1a**	10	BioRad PreCast TGX 4-15% gradient gel	PVDF	1:5000	MA3-912, ThermoFisher Scientific
**SERCA2a**	2.5	BioRad PreCast TGX 4-15% gradient gel	PVDF	1:5000	MA3-919, ThermoFisher Scientific
**RYR**	10	BioRad PreCast TGX 4-15% gradient gel	PVDF	1:2000	MA3-925, ThermoFisher Scientific
**p-RYR**	10	BioRad PreCast TGX 4-15% gradient gel	PVDF	1:2000	AF3703, Affinity Biotech
**4-HNE**	10	BioRad PreCast TGX 4-15% gradient gel	PVDF	1:5000	AB5605, Millipore Sigma
**SOD**	10	BioRad PreCast TGX 4-15% gradient gel	PVDF	1:5000	NB100-1992SS, Novus
**SLN**	25	Homemade tricine	Nitrocellulose	1:250	ABT13, Sigma Aldrich
**NNAT**	15	Homemade tricine	PVDF	1:1000	78122S, Cell Signaling Technology
**PLN**	10	Homemade tricine	PVDF	1:2000	MA3-922, ThermoFisher Scientific

Abbreviations: sarco(endo)plasmic reticulum Ca^{2+} ATPase (SERCA); ryanodine receptor (RYR); 4-hydroxynonenal (4-HNE); superoxide dismutase (SOD); sarcolipin (SLN); neuronatin (NNAT); phospholamban (PLN); polyvinylidene fluoride (PVDF)

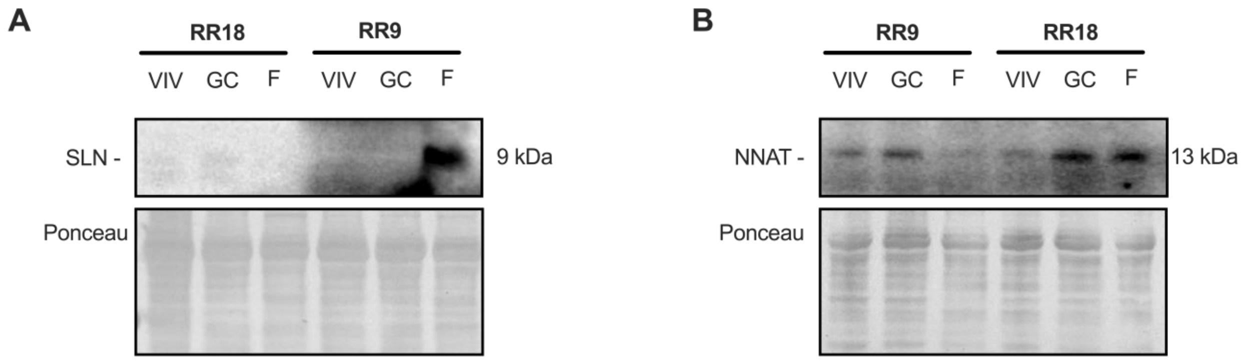

Supplemental Figure 1. Representative Western blots of SLN and NNAT in RR-9 vs RR-18 soleus. Representative images show differential responses to spaceflight between the RR-9 and RR-18 missions in SLN (**A**) and NNAT (**B**).
